# Hybrid Email and Outpatient Clinics to Optimize Maintenance Therapy in Acute Lymphoblastic Leukemia

**DOI:** 10.1097/MPH.0000000000002796

**Published:** 2023-12-12

**Authors:** Tushar Mungle, Ananya Mahadevan, Parag Das, Amit K. Mehta, Manash P. Gogoi, Bishwaranjan Jana, Niharendu Ghara, Debjani Ghosh, Vaskar Saha, Shekhar Krishnan

**Affiliations:** * Clinical Research Unit; † Department of Cell Biology; ‡ Tata Consultancy Services, Tata Translational Cancer Research Centre; § Department of Paediatric Haematology and Oncology, Tata Medical Center, Kolkata, India; ∥ Division of Cancer Sciences, Faculty of Biology, Medicine and Health, School of Medical Sciences, University of Manchester, Manchester, UK

**Keywords:** low–middle-income countries, acute lymphoblastic leukemia, maintenance therapy, e-clinic, electronic medical records

## Abstract

Acute lymphoblastic leukemia treatment includes an outpatient (OP)-based 2-year maintenance therapy (MT). Over an 8-year period, patients were transited from only OP to a hybrid e-clinic/OP-clinic model. Electronic and patient-held medical records of acute lymphoblastic leukemia patients 1 to 18 years old during MT were used to analyze demographics, drug doses, treatment response and cost. A survey evaluated family satisfaction with the hybrid service. Four hundred and seventy-eight children, all with at least 1 year of MT from March 13, 2014 to March 24, 2022 were grouped into 4 treatment eras, representing the transition from all OP (era 1) to the current hybrid MT practice (era 4). Cohort demographics were similar across all eras. With transition to era 4, OP visits decreased to a third (16 to 18/48 visits). Practice optimization in era 2 resulted in higher MT dose intensity in subsequent eras (era 1: median 82% [interquartile range, 63 to 97]; era 2: 93% [73 to 108]; era 3: 88% [68 to 106]; era 4: 90% [74 to 114], *P*<0·0001), with no differences in absolute neutrophil count or neutropenia-related toxicity (*P*=0.8). The hybrid service reduced MT expenses by ~50% and families (133/156, 85%) reported being very satisfied. Our experience indicates that a hybrid model is feasible, effective and less burdensome for patients and families.

Acute lymphoblastic leukemia (ALL) is the commonest cancer in childhood. Modern ALL treatment protocols treat intensively for 6 to 8 months, followed by 2 years of continuation or maintenance therapy (MT).^1^ During MT, children are treated with oral antimetabolite drugs: 6-mercaptopurine (6MP) administered daily, and methotrexate (MTX) administered weekly. 6MP, a prodrug, is metabolized by the salvage purine biosynthesis pathway to the pharmacologically active 6-thioguanine (TGN) nucleotides. Polymorphisms in genes encoding enzymes of the purine metabolic pathways lead to considerable individual variations in drug metabolite levels after systemic drug exposure. This affects efficacy and toxicity. The most common side effect is cytopenias.^2^ After treatment initiation, about 4 to 6 weeks of continuous oral antimetabolite therapy are required to obtain steady intracellular TGN levels.^2^ Subsequently, drug doses are require to be titrated individually to maintain steady TGN levels that are therapeutic but not toxic. This can be achieved by periodically monitoring levels of TGN incorporated in DNA of circulating leukocytes (DNA-TGN) and adjusting drug doses accordingly.^3^ Alternatively, red blood cell concentrations of 6MP metabolites such as TGN and 6-methylmercaptopurine can be measured,^4–6^ though this is less accurate for dose adjustments. DNA-TGN monitoring is not available widely, and levels that demarcate efficacy from toxicity are not yet known. Instead, blood counts are used as surrogate markers of antimetabolite drug exposure, and doses are adjusted to maximum tolerated doses that maintain safe low absolute neutrophil (ANC) and platelet counts.^7^ The aim is to avoid suboptimal antimetabolite dosing as this increases the risk of relapse.^8–10^

At the Tata Medical Center (TMC), Kolkata, a systematic MT program for children with ALL was initiated in late 2013. Initially, children visited the outpatient (OP) clinic every 2 weeks for a complete blood count test (CBC) and adjustment of 6MP and MTX doses. Patients maintained a printed record of serial CBC results and prescribed drug doses (see table, Supplemental Digital Content 1, http://links.lww.com/JPHO/A662) to facilitate treatment monitoring and dose titration. Over time, TMC began to receive patients from outside the state and from the neighboring countries of Bangladesh and Bhutan. For these patients, an email clinic service (e-clinic) was offered (started in 2017). Families emailed CBC reports and the clinical team responded with dosage recommendations. OP visits for these patients were scheduled every 3 months in conjunction with intrathecal chemotherapy treatments. By the end of 2018, a mixed mode of consultation (e-clinic and in-person) was being offered to all patients during MT. In mid-March 2020, the SARS-CoV-2 pandemic resulted in an enforced lockdown for many months. Patients were unable to travel, and hospital services were curtailed. All MT patients were moved to the e-clinic to ensure the continuation of therapy. We report on how this experience catalyzed the development of a hybrid approach to MT management at our center, decreasing the burden on families and the hospital while continuing to maintain drug dose titrations and optimal drug dosing.

## PATIENTS AND METHODS

### Patients

All children treated for ALL at TMC on the ICiCLe-ALL-14 protocol^11^ who commenced MT between March 13, 2014 and March 24, 2022 were eligible for the study. Patients without MT data for a minimum of 4 cycles starting from cycles 1 to 4 were not analyzed. Children were recruited with consent and with ethical approval (Institutional Review Board: EC/TMC/12/13).

### Treatment

**FIGURE 1:**
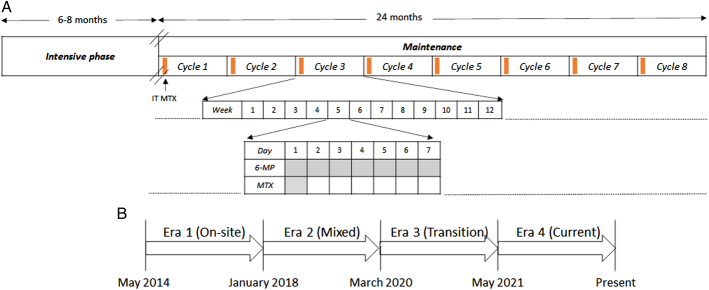
A, Acute lymphoblastic leukemia (ALL) treatment schematic for ICiCLe-ALL-14 protocol. B, Timeline for various eras. 6MP indicates 6-mercaptopurine; MTX, methotrexate.

In the ICiCLe-ALL-14 protocol, the intensive treatment phase lasts for 24 to 29 weeks depending on risk stratification (Fig. 1A).^11^ MT is divided into eight 12-week cycles for a total of 96 weeks. Together with daily oral 6MP and weekly MTX, children receive intrathecal MTX once every cycle and oral trimethoprim-sulphamethoxazole prophylaxis. As per protocol, a CBC is done every 2 weeks or more frequently if required. Overall, a minimum of 48 “visits” are required either as OP or a combination of e-clinic and OP visits. MT OP and e-clinics are held twice weekly, jointly by a consultant and a resident.

### Era Definition and Cohort Selection

For purposes of this study, the temporal changes in the delivery of MT were categorized into eras (Fig. 1B). In era 1 (May 1, 2014, to January 25, 2018) children receiving MT attended OP at least once in 2 weeks. In era 2 (January 26, 2018, to March 25, 2020) email consultations (e-clinic) were introduced, initially selectively for patients living far away from TMC and later for all patients. Era 3 (March 26, 2020, to May 14, 2021) marked the SARS-CoV-2 pandemic, requiring e-clinic MT management of all patients in this era as most patients were unable to travel to the hospital. The adaptations in era 3 led to the introduction of a hybrid MT service model in era 4 (May 15, 2021, to February 28, 2023) for all patients, consisting of a minimum of e-clinic and 2 in-person visits in each 12-week MT cycle. Era 4 comprises of 2 subgroups: patients who have completed MT and those who have completed a minimum 4 cycles of MT. Patients who were treated across eras were assigned to the era where >50% of MT was administered.

### Data Collection

Patient-held data sheets (see table, Supplemental Digital Content 1, http://links.lww.com/JPHO/A662) from eras 1 to 3 and e-clinic logbook entries from era 2, containing CBC and doses of 6MP and MTX, were digitized. Any missing data from handheld data sheets were completed by retrieval from electronic medical records. In era 3 and 4, a semiautomated spreadsheet (see spreadsheet, Supplemental Digital Content 2, http://links.lww.com/JPHO/A663) was created, individualized for each patient, stored on the hospital server, and accessible to both the e-clinic and OP teams. Data on MT information^12^ (see spreadsheet, Supplemental Digital Content 3, http://links.lww.com/JPHO/A664) were extracted from these records. Along with OP records, emails from patients were screened to check for additional comments by families. Hospital admissions and registered postal codes of the patients were extracted from electronic medical records. The postal code was used to identify the home address^13^ and compute the minimum distance between the patient’s home to the hospital.

### Dose and Response Analysis

In the ICiCLe-ALL-14 protocol, the recommended start doses of 6MP and oral MTX were 60 mg/m^2^/d and 20 mg/m^2^/wk, respectively (protocol-recommended doses). At each visit, drug doses were titrated based on blood counts. The targeted weighted means of prescribed antimetabolite doses over the course of MT were calculated for each patient and reported as a proportion of the protocol-recommended doses to represent dose intensities (DIs). DIs were determined individually for 6MP, MTX and for the antimetabolite combination (product of the DIs of 6MP and MTX). The interval (in weeks) between dose prescription visits (in-person or remotely) determined the weighted values^14^ (exemplar calculations provided in a spreadsheet, Supplemental Digital Content 4, http://links.lww.com/JPHO/A665). Neutropenia was defined as an ANC of ≤0.5×10^9^/L. Duration of neutropenic episodes refers to the interval (in weeks) for recovery of ANC from ≤0.5×10^9^/L to ≥0.75×10^9^/L. The median and interquartile ranges are used to report neutropenia episodes and the weighted mean of drug doses and drug DIs.

### Distance and Cost Analysis

The minimum cost incurred by each patient to travel the minimum distance to the hospital and back was computed at a cost of ₹3/km.^15^ The total minimum cost of MT was estimated based on the patient’s total OP visits, and the calculations were extended to include the family (1 child and 2 adults).

### Patient Satisfaction Survey

A simplified non-validated telephone survey was used to record the experience of families with the e-clinic MT service. The interview used a structured questionnaire (see survey, Supplemental Digital Content 5, http://links.lww.com/JPHO/A666) to evaluate user satisfaction, service preference (e-clinic alone, in-person alone or the era 4 hybrid model), and costs associated with hospital visits and email consultations. Telephone interviews were performed by members of the clinical team with interviews restricted to parents/caregivers who had accessed the e-clinic on at least 5 occasions, as well as accompanied patients during in-person visits.

### Statistical Analysis

Continuous variables are reported as the median interquartile range. Comparisons across groups were performed using the Kruskal-Wallis rank sum test, and pairwise comparisons were performed using the Wilcoxon rank sum test with the Bonferroni correction. Categorical variables are reported as absolute values and proportions (%). All statistical analyses were performed using R statistical software (version 4.1.3) and RStudio (version 2022.02.2). All statistical tests were 2 tailed with *P*<0.05 considered significant.

## RESULTS

A total of 478 patients were analyzed-163, 116, 68, and 131 in eras 1, 2, 3, and 4, respectively. Era 4 was subdivided into 2: 69 patients who received all 8 MT cycles and 62 patients with a minimum of 4 completed MT cycles. Patient characteristics are described in Table 1.

**TABLE 1:** Patient Characteristics

					Era 4		
	Whole cohort	Era 1	Era 2	Era 3	Overall	Completed MT	Completed ≥4 cycles
Year		May 2014 to January 25, 2018	January 26, 2018 to March 25, 2020	March 26, 2020 to May 14, 2021	May 15, 2021 to February 28, 2023	—	—
N	478	163	116	68	131	69	62
Age (y)							
Median [IQR]	4.7 [3.1-7.7]	4.7 (3.1-7.5)	4.9 [2.9-9.6]	4.5 [3.6-7]	4.9 [3.2-7.0]	4.7 [2.8-8.1]	5.1 [3.5-6.4]
Sex, n (%)							
Female	186 (39)	62 (38)	42 (36)	27 (40)	55 (42)	32 (46)	23 (37)
Male	292 (61)	101 (62)	74 (64)	41 (60)	76 (58)	37 (54)	39 (63)
Lineage, n (%)							
B	405 (85)	139 (85)	93 (80)	56 (82)	117 (89)	62(90)	55 (89)
T	73 (15)	24 (15)	23 (20)	12 (18)	14 (11)	7 (10)	7 (11)
WBC (N = 474)^*^							
Median [IQR]	16.3 [7.6-46.4]	14.8 [6.7-33.4]	23 [7.5-53.8]	12.3 [7.0-42.3]	17 [8.3-56.4]	12.4 [6.9-47.8]	25.3 [11.4-78.1]

* Presentation WBC data missing for 4 patients diagnosed outside but treated at TMC.

IQR indicates interquartile range; MT, maintenance therapy; WBC, white blood cell count at presentation (×10^9^/L).

### MT e-clinics Decrease Hospital Visits and OP Workload

In era 1, we did not offer a regular email clinic. Seventeen percent (28/163) of patients who lived further afield did send emails, but for most this was occasional, and the median emails sent per patient was 0 (0 to 0). In era 2, 72% (83/116) requested email clinics and the median was 10 (0 to 17) email consultations per patient. To handle the workload, personnel were designated to handle approximately 12 email consultations per week. In era 3, with the pandemic severely curtailing travel, all patients were managed through the e-clinic. A formal twice-weekly e-clinic was developed with a resident, consultant, and data manager dealing with a median 44 email visits per clinic. Post-pandemic in era 4, we formalized this as a hybrid service, offering patients 4 e-clinics, 1 OP visit, and 1 day-care visit for intrathecal MTX in each 12-week MT cycle. This equates to 32 e-clinics, 8 OP visits, and 8 day-care visits during MT. Patients who live closer to the hospital often choose to come to OP more regularly and some patients, either due to compliance or tolerance issues, require more frequent OP visits bringing the median number of email clinics per patient to 33 (30 to 37) (era 4 patients who completed MT) with 47 (35 to 52) emails per clinic (Table 2).

**TABLE 2:** Characteristics Data for e-clinic at TMC, Patients’ e-clinic Visit, and Mode of Consultation (email and OPD)

				Era 4		
	Era 1	Era 2	Era 3	Overall	Completed MT	Completed ≥4 cycles
Patients						
N	163	116	68	131	69	62
Patient who availed e-clinic, n (%)	28 (17)^*^	83 (72)	68 (100)	130 (99)	69 (100)	61 (98)
E-clinic consultations per patient†	0 [0-0]	10 [0-17]	31 [27-36]	29 [20-34]	33 [30-37]	20 [16-26]
Patients per e-clinic	—	12 [10-15]	44 [40-49]	47 [35-52]	—	—
Mode of consultation						
Direct						
n	135	58	39	70	37	31
E-clinic consultation	0 [0-0]	0 [0-6]	28 [20-30]	22 [17-26]	30 [28-32]	16 [14-18]
OP visits	44 [31-49]	48 [43-56]	21 [16-29]	17 [13-24]	18 [13-24]	16 [12-22]
Virtual						
n	28^*^	58	29	61	32	31
E-clinic consultation	3 [1-8]^*^	18 [14-28]	38 [34-40]	34 [32-38]	38 [35-42]	26 [24-30]
OP visits	32 [20-41]^*^	26 [21-34]	14 [11-17]	15 [12-19]	18 [13-22]	14 [12-18]

Values represent median [interquartile range] unless stated otherwise.

“Direct” refers to patients whose e-clinic consultations were ≤the median e-clinic consultations per patient† for the respective era.

“Virtual” refers to patients whose e-clinic consultations were >the median e-clinic consultations per patient† for the respective era.

* Some patients in the later months of era 1 had availed email service.

MT, maintenance therapy; OP, outpatient.

To better understand the adoption of email clinics by patients, we divided patients in each era into 2 cohorts, a “virtual” cohort, whose number of e-clinic visits was above the median in the respective era, and a “direct” cohort whose number of e-clinic visits were below or equaled the median in that era. As shown in Table 2, of the expected 48 clinic visits in era 4, “virtual” patients recorded a median 56 visits (e-clinics: 38 [35 to 42]; in-person visits: 18 [13 to 22]), with the additional visits: required for weekly supervision during neutropenia episodes. “Direct” patients recorded a 4:2 ratio of e-clinic to OP visits (median e-clinics: 30 [28 to 32]; median in-patient visits: 18 [13-24]) highlighting that these patients too were using the e-clinic more often than OP reviews. With the frequency of OP visits halved, the number of patients in each OP clinic reduced considerably. The OP clinic now sees 40 to 50 MT patients each time, down from 70 to 80 patients per clinic in eras 1 to 2.

### Improved Dose Optimization With e-clinic and Hybrid Clinics

For each era, the weighted mean DIs of 6MP and MTX and the weighted mean ANC over the entire course of MT for each patient were calculated to determine the median DIs and ANCs in each era cohort. DIs were significantly lower in era 1 compared with eras 2 to 4 (*P*=0.0004 and <0.0001 for 6MP and MTX DIs, respectively) (Table 3). Dose titration practice was optimized in era 2, and as a consequence, 6MP and MTX DIs increased significantly (*P*=0.0009 and <0.0001, respectively, see table, Supplemental Digital Content 6, http://links.lww.com/JPHO/A667). These improvements in DIs were maintained in era 3 despite the COVID-19 pandemic but with wider DI variations since MT clinics were less frequent due to pandemic restrictions. These variations stabilized in era 4 as clinics were regularized and families traveled or emailed for MT dose consultations. Overall, there were no differences in DIs during eras 2 to 4 (*P*=0.43 and 0.34 for 6MP and MTX DIs, respectively). Despite the increase in drug DIs, the median weighted mean ANCs did not differ significantly across the eras (Table 3). Optimization of dose titration in era 2 was accompanied by an increase in the frequency and duration of neutropenia episodes (*P*=0.0046 and 0.0286, respectively) compared with era 1 (see table, Supplemental Digital Content 6, http://links.lww.com/JPHO/A667), but declined in eras 3 and 4 (Table 3). There were no differences in DIs and neutropenia episodes between the “virtual” and “direct” groups (see table, Supplemental Digital Content 7, http://links.lww.com/JPHO/A668).

**TABLE 3:** Treatment and Treatment Response in Different MT Eras

	Era 1	Era 2	Era 3	Era 4 (MT complete)	*P* ^*^ (overall)	*P* ^†^ (eras 2, 3, 4)
wm6MP dose intensity (%)	82 [63-97]	93 [73-108]	88 [68-106]	90 [74-114]	0.0004	0.43
wmMTX dose intensity (%)	81 [67-94]	95 [77-110]	93 [69-108]	94 [76-114]	<0.0001	0.34
wmANC (× 109/L)	1.8 [1.5-2.1]	1.8 [1.5-2.1]	1.7 [1.5-2.1]	1.8 [1.5-2.1]	0.8	0.64
Neutropenia episodes (n)	1 [0-3]	2 [1-3]	1 [1-2]	2 [1-2]	0.0019	0.0027
Neutropenia duration (weeks)	3 [0-5]	4 [2-7]	3 [2-5]	3 [1-4]	0.0059	0.0038

Values represent median [interquartile range] unless stated otherwise.

Weight, interval (in weeks) between clinic (outpatient-/e-) visits; dose intensity, (prescribed dose ÷ protocol dose)%.

Neutropenia, ANC ≤0.5×10^9^/L. Recovery from neutropenic state when ANC ≥0.75×10^9^/L.

Neutropenia duration (weeks), interval between onset of neutropenia and ANC recovery, cumulative all neutropenia episodes.

* Kruskal-Wallis rank sum test comparing all 4 eras.

† The latter 3 eras.

6MP indicates 6-mercaptopurine; ANC, absolute neutrophil count; MT, maintenance therapy; MTX, methotrexate; wm, weighted mean.

With the hybrid approach in era 4, drug DIs were increased without an increase in hematological toxicities. The wmANC was 1.8 (1.5 to 2.1) while the target ANC was between 1.5 to 0.75, suggesting room for further dose optimization (based on the “treat to tolerance” principle). The increased hematological toxicity in era 2 reflects a learning curve with dose titration practice and structured monitoring. Our experience suggests that parents are quick to inform complications or toxicities by email (see table, Supplemental Digital Content 8, http://links.lww.com/JPHO/A669) and as a result, are seen in the clinic if required.

### E-clinics Decrease MT Treatment Costs

We hypothesized that families’ preference for e-clinics was related to the distance they had to travel for OP visits. Residence postal codes were available for 332/478 (70%) patients who had completed MT. With the widening of the catchment area, the median distance traveled to the hospital increased from eras 1 to 4 (see table a, Supplemental Digital Content 9, http://links.lww.com/JPHO/A670). In era 4, families who lived within a median of 48 km from the hospital were more likely to opt for in-person appointments. For families living within a 100 km range, the availability of public transport allows clinic attendance as a day visit. Families residing beyond this distance needed to spend at least 1 night in the city when attending MT clinics. From era 2 onward, families living ≥100 km were all choosing e-clinics (see table b, Supplemental Digital Content 9, http://links.lww.com/JPHO/A670).

The current era 4 hybrid approach entails an ~50% decrease in OP costs as the cumulative OP visits over the course of MT decreased to 16 in era 4 compared with the earlier 48 OP visits (Table 4). The decrease in OP visits in era 4 results in aggregate costs savings of ~₹18,000 (~£180), considering that 1 OP visit during MT costs ₹560 (~£5.50, including cost of the CBC test [₹410, ~£4.00] and OP consultation charge [₹150, ~£1.50]) (see tables a-c, Supplemental Digital Content 10, http://links.lww.com/JPHO/A671).

**TABLE 4:** OP Visit Cost Comparisons Between MT Eras 1 and 4

	Era 1		Era 4 (completed MT subgroup)	
	N (%)	Cost incurred^*^,^†^	N (%)	Cost incurred^*^,^†^
Distance, home to hospital (km)				
<50	59 (44)	20.2 [14.6-28.1]	22 (37)	8.8 [5.2-11.7]
≥50 and <100	25 (19)	58 [44.5-70.4]	3 (5)	21.6 [15.9-26.7]
≥100 and <150	14 (10)	87.6 [57-103]	7 (12)	46 [42.5-57.6]
≥150	37 (27)	161.2 [120.4-227.9]	27 (46)	83 [64.6-112.2]

* Minimum total OP visit costs in MT, assuming 3 people (1 child, 2 adults) used public transport (fare, ₹3/km).

† Values refer to median [interquartile range].

km indicates kilometer; MT, maintenance therapy.

### The Hybrid MT Service Elicits Favorable Feedback From Patients and Families

A telephone survey was carried out with a pre-tested questionnaire to assess the acceptance of the hybrid clinic format; 156/199 (78%) families from eras 3 and 4 responded (Table 5). The majority of families (133/156, 85%) were very satisfied with the MT clinic format and felt that the advice received was timely and all additional queries were answered. There were no reported issues in understanding the advice sent by email. Nearly a third of families reported that they would be happier with more (ie, 3) OP visits per cycle, and no family wanted a full e-clinic–based MT service. Nearly all families reported that OP visits were more stressful and disrupted the child’s schooling and social life and the majority agreed that the introduction of e-clinics was consequently less demanding on the child and families.

**TABLE 5:** Survey Responses, MT e-clinic User Experience and Satisfaction

N= 156	Response
Are user satisfied with the email service, n (%)	
1. Not at all	—
2. No	—
3. Moderately satisfied	23 (15)
4. Satisfied or 5. Very satisfied	133 (85)
Receive timely advice? (Yes)	156 (100)
Is response understandable? (Yes)	155 (99.4)
Are your queries answered? (Yes)	153 (98)
What kind of MT service would you prefer? n (%)	
1. Email clinic alone	11 (7)
2. OP visits alone	2 (1)
3. Three email clinics + 3 OP visits (per MT cycle)	44 (28)
4. Four email clinics + 2 OP visits (per MT cycle)^*^	81 (52)
5. Five email clinics + 1 OP visit (per MT cycle)	18 (12)
Which is less stressful for you and your child?	
Email clinic	155 (99)
OP clinic	1 (1)
Which is more beneficial for your child’s regular school?	
Email clinic	156 (100)
OP clinic	0
Which is better for you and your child’s physical health?	
Email clinic	128 (82)
OP clinic	28 (18)
Which is more beneficial for you and your child’s social life?	
Email clinic	153 (98)
OP clinic	3 (2)

* Current practice, OP visits include 1 clinic visit and 1 day case visit for intrathecal chemotherapy.

MT indicates maintenance therapy; OP, outpatient; TMC, Tata Medical Center cancer hospital, Kolkata, India.

## DISCUSSION

The period of MT in childhood ALL lasts for around 2 years during which dosing is personalized for each patient based on serial blood counts. Over the last decade, we have worked to develop a system to create a simplified and standardized approach acceptable to patients and medical staff. In era 1, we introduced patient-held records of doses prescribed and the accompanying blood counts entered by hand at each clinic visit (see spreadsheet, Supplemental Digital Content 2, http://links.lww.com/JPHO/A663). Patients came with this record each time to the clinic. Gradually e-clinics (era 2) were introduced for families residing far away from hospital. During the pandemic (era 3), with patients unable to come to the hospital, we created digitized sheets, which are present on our server and contain past and current records. In era 4, these sheets are filled in the e-clinic and in the OP clinic, with patients receiving a printed-out copy whenever they come to the hospital. The e-clinics progressed from contributing only one-fifth to two-thirds of the total MT visits over the years and is the standard norm for ALL patients at TMC. Since January 2021, there are twice weekly e-clinics. With no missing data and careful scrutiny, the weighted mean dose was intensified without an increase in toxicity. The telephonic survey was conducted by the clinical team known to the families, potentially introducing a favorable bias. Nevertheless, results of the telephone survey suggest that families greatly preferred the hybrid approach, though some recommended also having a 24-hour dedicated hotline for MT.

A major concern for patients in low–middle-income countries is the financial toxicity during cancer therapy.^16,17^ We have previously reported that OP costs constitute 40% to 60% of ALL treatment expense,^18^ and with the current hybrid model, the OP visit cost is reduced by ~50%. Subsequently, we believe Out-of-pocket expenses for food and lodging incurred during hospital visits will be reduced.

In the past, efforts have been made to study and assess the feasibility and safety of remote MT consultations using small cohorts over short durations.^19–21^ Although successful at TMC, the hybrid approach presents certain challenges. E-clinics require additional manpower and time allocation. While no patient refused email clinic, patients from neighboring countries were occasionally irregular with emails. We sent all emails and prescriptions in English. Families replied in English or in vernacular. Many families used internet-cafés to email the blood reports. To date, we have not encountered any misinterpretation of instructions, and families report the assistance of other family members or internet-café employees when required. If there is a doubt, clinical unit members have called parents to clarify. Medication adherence is a critical factor during MT.^22,23^ Though this does not appear to be a major problem at our center, this is difficult to diagnose without a clinic visit. It is possible that some toxicities such as hypoglycemia may be under-reported by parents.^19^ We educate parents on the expected toxicities at the start of MT. These are reported by parents over email and addressed immediately or in the next OP clinic. One challenge we did face was the reliability of blood counts done elsewhere due to varying lab standards. This affected a few patients whose counts done locally differed widely from those done at TMC. In all cases, we were able to resolve this quickly by working with the families to identify alternative laboratories. Chemotherapy drugs are not widely available in small rural areas and patients are prescribed sufficient medication to take home with them when they visit OP. During the pandemic, this did pose a problem for some patients who were unable to procure supplies locally, though distributors did try their best to directly deliver to their homes.

The digitized longitudinal data eases optimal dose titration practice. In the future, additional information can be included to assist in treating inherent drug-sensitive patients with protocol modulation^3^ or adjuvant therapy.^24^ The system developed to support the e-clinic can be adapted to clinical decision-making software.^25–27^ This will facilitate the standardization of MT therapy across centers in countries the size of India. Patients can upload the CBC report and receive back drug prescriptions with a possible visual aid of daily dose distribution and a reminder message on their mobile phones to take the drugs. With these data stored on a database, machine learning/artificial intelligence tools can further optimize dosing^28^ and aid clinicians.

## Supplementary Material
