# Supplementary material for: Hybrid Email and Outpatient Clinics to Optimize Maintenance Therapy in Acute Lymphoblastic Leukemia
Source: J Pediatr Hematol Oncol. 2023 Dec 12;46(1):39–45. doi: 10.1097/MPH.0000000000002796 (PMC10756697; doi:10.1097/MPH.0000000000002796)
Supplement: Supplementary file 1 [file mph-46-039-s001.pdf]

TMC KIDS

ICiCLe ALL 14 Maintenance Schedule

Maintenance Cycle	
Patient Weight (kg)	
Patient Height (cm)	
SA (m^2)	

Name **Date of Birth** **MR No**

		Week 1	Week 2	Week 3	Week 4	Week 5	Week 6	Week 7	Week 8	Week 9	Week 10	Week 11	Week 12
	Date												
	Neutrophil Count												
	Platelet Count												
Drug	Dose (100%)	Weekly Dose											
6-Mercaptopurine (60mg/m2)													
Methotrexate (20mg/m2)													
IT Methotrexate													
Cotrimoxazole													

Cotrimoxazole
$<0.75m^2$ = 240mg
≥ 0.75 - $<1m^2$ = 360mg
$\geq 1m^2$ = 480mg

Maintain ANC between 750 and 1500.
If ≥500 but <750, decrease dose to 50%.
If <500, stop drugs, consider weekly count check and restart once ≥750
If counts have been low for 4 weeks without therapy, stop Cotrimoxazole
If ANC >1500 for 4 weeks, increase 6-MP by 25%.
If no change over 4 weeks, increase MTX by 25%. If still no change, consider non compliance
Maintain platelet count over 75,000.
If ≥50,000 but <75,000 decrease dose to 50%.
If <50,000, stop drugs and restart once ≥75,000
If only platelet counts are low, consider Veno-occlusive disease
AVOID ORAL METHOTREXATE IN WEEK WHEN IT METHOTREXATE IS GIVEN
Email Id: tmckids.maintenance@tmckolkata.com **PH#**: 033-66057090
