# Supplementary material for: Hybrid Email and Outpatient Clinics to Optimize Maintenance Therapy in Acute Lymphoblastic Leukemia
Source: J Pediatr Hematol Oncol. 2023 Dec 12;46(1):39–45. doi: 10.1097/MPH.0000000000002796 (PMC10756697; doi:10.1097/MPH.0000000000002796)
Supplement: Supplementary file 3 [file mph-46-039-s003.docx]

**SDC 3**: Patients (de-identified) id whose data was used in the study.

| UPN_1 | UPN_31 | UPN_61 | UPN_91 | UPN_121 | UPN_151 | UPN_181 | UPN_222 | UPN_261 | UPN_291 | UPN_321 | UPN_351 | UPN_381 | UPN_411 | UPN_441 | UPN_471 |
| --- | --- | --- | --- | --- | --- | --- | --- | --- | --- | --- | --- | --- | --- | --- | --- |
| UPN_2 | UPN_32 | UPN_62 | UPN_92 | UPN_122 | UPN_152 | UPN_182 | UPN_223 | UPN_262 | UPN_292 | UPN_322 | UPN_352 | UPN_382 | UPN_412 | UPN_442 | UPN_472 |
| UPN_3 | UPN_33 | UPN_63 | UPN_93 | UPN_123 | UPN_153 | UPN_183 | UPN_224 | UPN_263 | UPN_293 | UPN_323 | UPN_353 | UPN_383 | UPN_413 | UPN_443 | UPN_473 |
| UPN_4 | UPN_34 | UPN_64 | UPN_94 | UPN_124 | UPN_154 | UPN_184 | UPN_225 | UPN_264 | UPN_294 | UPN_324 | UPN_354 | UPN_384 | UPN_414 | UPN_444 | UPN_474 |
| UPN_5 | UPN_35 | UPN_65 | UPN_95 | UPN_125 | UPN_155 | UPN_185 | UPN_226 | UPN_265 | UPN_295 | UPN_325 | UPN_355 | UPN_385 | UPN_415 | UPN_445 | UPN_475 |
| UPN_6 | UPN_36 | UPN_66 | UPN_96 | UPN_126 | UPN_156 | UPN_188 | UPN_228 | UPN_266 | UPN_296 | UPN_326 | UPN_356 | UPN_386 | UPN_416 | UPN_446 | UPN_476 |
| UPN_7 | UPN_37 | UPN_67 | UPN_97 | UPN_127 | UPN_157 | UPN_189 | UPN_229 | UPN_267 | UPN_297 | UPN_327 | UPN_357 | UPN_387 | UPN_417 | UPN_447 | UPN_477 |
| UPN_8 | UPN_38 | UPN_68 | UPN_98 | UPN_128 | UPN_158 | UPN_190 | UPN_231 | UPN_268 | UPN_298 | UPN_328 | UPN_358 | UPN_388 | UPN_418 | UPN_448 | UPN_478 |
| UPN_9 | UPN_39 | UPN_69 | UPN_99 | UPN_129 | UPN_159 | UPN_191 | UPN_233 | UPN_269 | UPN_299 | UPN_329 | UPN_359 | UPN_389 | UPN_419 | UPN_449 | UPN_479 |
| UPN_10 | UPN_40 | UPN_70 | UPN_100 | UPN_130 | UPN_160 | UPN_192 | UPN_234 | UPN_270 | UPN_300 | UPN_330 | UPN_360 | UPN_390 | UPN_420 | UPN_450 | UPN_480 |
| UPN_11 | UPN_41 | UPN_71 | UPN_101 | UPN_131 | UPN_161 | UPN_194 | UPN_235 | UPN_271 | UPN_301 | UPN_331 | UPN_361 | UPN_391 | UPN_421 | UPN_451 | UPN_481 |
| UPN_12 | UPN_42 | UPN_72 | UPN_102 | UPN_132 | UPN_162 | UPN_196 | UPN_237 | UPN_273 | UPN_302 | UPN_332 | UPN_362 | UPN_392 | UPN_422 | UPN_452 | UPN_482 |
| UPN_13 | UPN_43 | UPN_73 | UPN_103 | UPN_133 | UPN_163 | UPN_197 | UPN_238 | UPN_274 | UPN_303 | UPN_333 | UPN_363 | UPN_393 | UPN_423 | UPN_453 | UPN_483 |
| UPN_14 | UPN_44 | UPN_74 | UPN_104 | UPN_134 | UPN_164 | UPN_199 | UPN_239 | UPN_275 | UPN_304 | UPN_334 | UPN_364 | UPN_394 | UPN_424 | UPN_454 | UPN_484 |
| UPN_15 | UPN_45 | UPN_75 | UPN_105 | UPN_135 | UPN_165 | UPN_200 | UPN_240 | UPN_276 | UPN_305 | UPN_335 | UPN_365 | UPN_395 | UPN_425 | UPN_455 | UPN_485 |
| UPN_16 | UPN_46 | UPN_76 | UPN_106 | UPN_136 | UPN_166 | UPN_201 | UPN_241 | UPN_277 | UPN_306 | UPN_336 | UPN_366 | UPN_396 | UPN_426 | UPN_456 | UPN_486 |
| UPN_17 | UPN_47 | UPN_77 | UPN_107 | UPN_137 | UPN_167 | UPN_203 | UPN_243 | UPN_278 | UPN_307 | UPN_337 | UPN_367 | UPN_397 | UPN_427 | UPN_457 | UPN_487 |
| UPN_18 | UPN_48 | UPN_78 | UPN_108 | UPN_138 | UPN_168 | UPN_204 | UPN_245 | UPN_279 | UPN_308 | UPN_338 | UPN_368 | UPN_398 | UPN_428 | UPN_458 | UPN_488 |
| UPN_19 | UPN_49 | UPN_79 | UPN_109 | UPN_139 | UPN_169 | UPN_205 | UPN_246 | UPN_280 | UPN_309 | UPN_339 | UPN_369 | UPN_399 | UPN_429 | UPN_459 | UPN_489 |
| UPN_20 | UPN_50 | UPN_80 | UPN_110 | UPN_140 | UPN_170 | UPN_206 | UPN_247 | UPN_281 | UPN_310 | UPN_340 | UPN_370 | UPN_400 | UPN_430 | UPN_460 | UPN_490 |
| UPN_21 | UPN_51 | UPN_81 | UPN_111 | UPN_141 | UPN_171 | UPN_207 | UPN_248 | UPN_283 | UPN_311 | UPN_341 | UPN_371 | UPN_401 | UPN_431 | UPN_461 | UPN_491 |
| UPN_22 | UPN_52 | UPN_82 | UPN_112 | UPN_142 | UPN_172 | UPN_208 | UPN_249 | UPN_284 | UPN_312 | UPN_342 | UPN_372 | UPN_402 | UPN_432 | UPN_462 | UPN_492 |
| UPN_23 | UPN_53 | UPN_83 | UPN_113 | UPN_143 | UPN_173 | UPN_209 | UPN_250 | UPN_285 | UPN_313 | UPN_343 | UPN_373 | UPN_403 | UPN_433 | UPN_463 | UPN_493 |
| UPN_24 | UPN_54 | UPN_84 | UPN_114 | UPN_144 | UPN_174 | UPN_211 | UPN_251 | UPN_288 | UPN_314 | UPN_344 | UPN_374 | UPN_404 | UPN_434 | UPN_464 | UPN_494 |
| UPN_25 | UPN_55 | UPN_85 | UPN_115 | UPN_145 | UPN_175 | UPN_212 | UPN_252 | UPN_289 | UPN_315 | UPN_345 | UPN_375 | UPN_405 | UPN_435 | UPN_465 | UPN_495 |
| UPN_26 | UPN_56 | UPN_86 | UPN_116 | UPN_146 | UPN_176 | UPN_213 | UPN_253 | UPN_290 | UPN_316 | UPN_346 | UPN_376 | UPN_406 | UPN_436 | UPN_466 | UPN_496 |
| UPN_27 | UPN_57 | UPN_87 | UPN_118 | UPN_147 | UPN_177 | UPN_214 | UPN_254 |  | UPN_317 | UPN_347 | UPN_377 | UPN_407 | UPN_437 | UPN_467 | UPN_497 |
| UPN_28 | UPN_58 | UPN_88 | UPN_119 | UPN_148 | UPN_178 | UPN_215 | UPN_255 |  | UPN_318 | UPN_348 | UPN_378 | UPN_408 | UPN_438 | UPN_468 | UPN_498 |
| UPN_29 | UPN_59 | UPN_89 | UPN_120 | UPN_149 | UPN_179 | UPN_216 | UPN_256 |  | UPN_319 | UPN_349 | UPN_379 | UPN_409 | UPN_439 | UPN_469 | UPN_499 |
| UPN_30 | UPN_60 | UPN_90 |  | UPN_150 | UPN_180 | UPN_217 | UPN_258 |  | UPN_320 | UPN_350 | UPN_380 | UPN_410 | UPN_440 | UPN_470 | UPN_500 |
|  |  |  |  |  |  | UPN_219 | UPN_259 |  |  |  |  |  |  |  |  |
|  |  |  |  |  |  |  | UPN_260 |  |  |  |  |  |  |  |  |

*Note*: Please visit https://data.mendeley.com/datasets/775hs9wrb5/1 for maintenance therapy data on UPNs mentioned in the SDC 3. (Reference: Mungle T, Mahadevan A, Gogoi MP, et al. Acute lymphoblastic leukaemia maintenance therapy dataset. 2023; 1. DOI:10.17632/775hs9wrb5.1)
