# Supplementary material for: Hybrid Email and Outpatient Clinics to Optimize Maintenance Therapy in Acute Lymphoblastic Leukemia
Source: J Pediatr Hematol Oncol. 2023 Dec 12;46(1):39–45. doi: 10.1097/MPH.0000000000002796 (PMC10756697; doi:10.1097/MPH.0000000000002796)
Supplement: Supplementary file 5 [file mph-46-039-s005.pdf]

# E-mail Maintenance Dose Advice Service

A survey to investigate e-mail services adopted for maintenace clinic

1. Study Number
   (Serial Number) *

OPD Maintenance Clinic

2. How much money do you spend for all the family people in **travelling** to Tata Medical Center for **one OPD** during maintenace therapy? *

- ○ ₹ 0-500
- ○ ₹ 501-1000
- ○ ₹ 1001-1500
- ○ ₹ 1501-2000
- ○ ₹ 2001 and above

During **one E-mail clinic**, how much money do you spend on

3. Travel *

₹ 0	₹ 1-500	₹ 501-1000	₹ 1001-1500	₹ 1501 and above
○	○	○	○	○

4. Blood Test *

₹ 0-200	₹ 201-400	₹ 401-600	₹ 601-800	₹ 801 and above
○	○	○	○	○

5. Emailing the blood report to Tata Medical Center from internet cafe or personal computer/mobile *

₹ 0	₹ 1-50	₹ 51-100	₹ 101-150	₹ 151 and above
○	○	○	○	○

6. Are you satisfied with the E-mail clinic dose advice service?

(**1 star** - Not at all; **2 star** - No; **3 star** - Average; **4 star** - Happy, **5 star** - Very Happy) *

☆ ☆ ☆ ☆ ☆

7. How can we improve the E-mail clinic dose advice service? *

8. Do you receive advice within 1 week of sending the e-mail? *

- ◯ Yes
- ◯ No

9. Is the advice sent during E-mail clinic understandable? *

- ◯ Yes
- ◯ No

10. If above answer (question 9) is no, please provide suggestions *

11. Are your queries answered during E-mail clinic? *

- ◯ Yes
- ◯ No

12. For 2 years of maintenance therapy, which one do you prefer? *

- ◯ Only E-mail clinic
- ◯ Only OPD at Tata Medical Center
- ◯ 3 E-mail clinic + 3 Hospital Visits (per cycle)
- ◯ 4 E-mail clinic + 2 Hospital Visits (per cycle) - currently practiced
- ◯ 5 E-mail clinic + 1 Hospital Visits (per cycle)

13. Which is less stressful for you and your child? *

- ◯ OPD Clinic
- ◯ E-mail Clinic

14. Which is more beneficial for your child's regular school? *

- ◯ OPD Clinic
- ◯ E-mail Clinic

15. Which is better for you and your child's physical health? *

- ◯ OPD Clinic
- ◯ E-mail Clinic

16. Which is more beneficial for you and your child's social life? *

- ◯ OPD Clinic
- ◯ E-mail Clinic
