# Supplementary material for: Hybrid Email and Outpatient Clinics to Optimize Maintenance Therapy in Acute Lymphoblastic Leukemia
Source: J Pediatr Hematol Oncol. 2023 Dec 12;46(1):39–45. doi: 10.1097/MPH.0000000000002796 (PMC10756697; doi:10.1097/MPH.0000000000002796)
Supplement: Supplementary file 6 [file mph-46-039-s006.docx]

| **SDC 6**. Statistical comparison of neutropenia episodes and duration between eras | | | | |
| --- | --- | --- | --- | --- |
|  | **Era 1** | **Era 2** | **Era 3** | **Era 4** |
|  |  |  |  | MT complete |
| **Weighted mean 6-MP** (p-values*) | | | | |
| Era 1 | - | 0.0009 | 0.4776 | 0.0076 |
| Era 2 |  | - | 1 | 1 |
| Era 3 |  |  | - | 1 |
| Era 4 |  |  |  | - |
| **Weighted mean MTX** (p-values*) | | | | |
| Era 1 | - | <0.0001 | 0.024 | <0.0001 |
| Era 2 |  | - | 1 | 1 |
| Era 3 |  |  | - | 1 |
| Era 4 |  |  |  | - |
| **Neutropenia Episodes** (p-values*) | | | | |
| Era 1 | - | 0.0046 | 1 | 1 |
| Era 2 |  | - | 0.17 | 0.0484 |
| Era 3 |  |  | - | 1 |
| Era 4 |  |  |  | - |
| **Neutropenia Duration** (p-values*) | | | | |
| Era 1 | - | 0.0286 | 1 | 1 |
| Era 2 |  | - | 0.2456 | 0.0063 |
| Era 3 |  |  | - | 1 |
| Era 4 |  |  |  | - |
| **Admissions** (p-values*) |  |  |  |  |
| Era 1 | - | 0.554 | 0.0011 | 0.0935 |
| Era 2 |  | - | 0.0946 | 1 |
| Era 3 |  |  | - | 1 |
| Era 4 |  |  |  | - |
| *Pairwise Wilcox test with bonferroni correction | |  |  |  |
