# Supplementary material for: Hybrid Email and Outpatient Clinics to Optimize Maintenance Therapy in Acute Lymphoblastic Leukemia
Source: J Pediatr Hematol Oncol. 2023 Dec 12;46(1):39–45. doi: 10.1097/MPH.0000000000002796 (PMC10756697; doi:10.1097/MPH.0000000000002796)
Supplement: Supplementary file 7 [file mph-46-039-s007.docx]

| **SDC 7**. Treatment and treatment response in sub-cohort’s e-mail and in-person | | | | |  |
| --- | --- | --- | --- | --- | --- |
|  | **Era 1** | **Era 2** | **Era 3** | **Era 4** | **p-value*** |
|  |  |  |  | (Completed MT) |  |
| **wm6-MP dose intensity** |  |  |  |  |  |
| Overall | 82 [63-97] | 93 [73-108] | 88 [68-106] | 90 [74-114] | 0.0004 |
| Direct | 82 [63-97] | 92 [72-111] | 75 [60-101] | 89 [71-117] |  |
| Virtual | 82 [71-94] | 94 [78-104] | 95 [81-106] | 91 [75-114] |  |
| p-value** | 0.6 | 0.8 | 0.016 | >0.9 |  |
| **wmMTX dose intensity** |  |  |  |  |  |
| Overall | 81 [67-94] | 95 [77-110] | 93 [69-108] | 94 [76-114] | <0.0001 |
| Direct | 81 [67-98] | 99 [76-117] | 86 [60-111] | 95 [76-115] |  |
| Virtual | 80 [67-97] | 95 [78-105] | 95 [81-105] | 93 [78-110] |  |
| p-value** | 0.6 | 0.5 | 0.15 | 0.6 |  |
| **wmANC (10^6/L)** |  |  |  |  |  |
| Overall | 1.8 [1.5-2.1] | 1.8 [1.5-2.1] | 1.7 [1.5-2.1] | 1.8 [1.5-2.1] | 0.8 |
| Direct | 1.8 [1.5-2] | 1.9 [1.6-2.2] | 1.7 [1.5-2.2] | 1.9 [1.7-2.2] |  |
| Virtual | 2 [1.7-2.4] | 1.7 [1.5-2] | 1.7 [1.5-1.9] | 1.7 [1.4-2] |  |
| p-value** | 0.009 | 0.11 | 0.3 | 0.06 |  |
| **Total Neutropenia Episodes** |  |  |  |  |  |
| Overall | 1 [0-3] | 2 [1-3] | 1 [1-2] | 2 [1-2] | 0.0019 |
| Direct | 2 [0-3] | 2 [1-3] | 1 [1-2] | 2 [1-2] |  |
| Virtual | 1 [0-3] | 2 [1-3] | 1 [1-2] | 2 [1-2] |  |
| p-value** | 0.4 | 0.6 | 0.8 | 0.8 |  |
| **Total Neutropenia Duration (weeks)** |  |  |  |  |  |
| Overall | 3 [0-5] | 4 [2-7] | 3 [2-5] | 3 [1-4] | 0.0058 |
| Direct | 3 [0-5] | 4 [2-6] | 3 [2-6] | 3 [1-5] |  |
| Virtual | 3 [0-5] | 5 [2-7] | 3 [2-5] | 3[1-4] |  |
| p-value** | 0.7 | 0.4 | 0.8 | 0.9 |  |
| **Admissions** |  |  |  |  |  |
| Number of patients (%) | 106 (65) | 69 (59) | 28 (41) | 35 (51) |  |
| Median [IQR] | 1 [0-2] | 1 [0-1] | 0 [0-1] | 1 [0-1] | 0.00087 |
| Values represent Median [Interquartile range] unless stated otherwise; | | | | | |
| Dose intensity is defined as a ratio of the dose prescribed on the given visit to the protocol recommended dose. The ratio was converted to percentage. Subsequently, weighted mean was computed (Supplementary Table SDC 3) | | | | | |
| Neutropenia is defined as ANC <= 0.5 (10^9/L). A patient recovers from neutropenic condition when ANC >0.75 x 10^9/L | | | | | |
| "Direct" is defined as patients who have fewer or an equal number of e-clinic consultations than the median e-clinic consultations/patient^ for the respective era | | | | | |
| "Virtual" is defined as patients who have more e-clinic consultations than the median e-clinic consultations/patient^ for the respective era | | | | | |
| *Kruskal-Wallis rank sum test; **Wilcoxon rank sum test to compare parameters from Direct and Virtual cohort | | | | | |
