# Supplementary material for: Hybrid Email and Outpatient Clinics to Optimize Maintenance Therapy in Acute Lymphoblastic Leukemia
Source: J Pediatr Hematol Oncol. 2023 Dec 12;46(1):39–45. doi: 10.1097/MPH.0000000000002796 (PMC10756697; doi:10.1097/MPH.0000000000002796)
Supplement: Supplementary file 8 [file mph-46-039-s008.docx]

| **SDC 8**. Complications reported by families via e-mail for current era cohort | | | |
| --- | --- | --- | --- |
|  | **Era 4** | | |
|  | Overall | Completed MT | Completed minimum 4 cycles |
| **N** | 131 | 69 | 62 |
| **Number of families [N (%)]** |  |  |  |
| Fever | 36 (27) | 22 (32) | 14 (23) |
| Cough and Cold | 31 (24) | 18 (26) | 13 (21) |
| Vomiting | 14 (11) | 8 (12) | 6 (10) |
| Pain | 18 (14) | 13 (19) | 5 (8) |
| Skin Irritation | 12 (9) | 7 (10) | 5 (8) |
| **Median [IQR] of reported complications** |  |  |  |
| Fever | 1 [1-2] | 1 [1-2] | 1 [1-1] |
| Cough and Cold | 1 [1-2] | 1 [1-1] | 1 [1-2] |
| Vomiting | 1 [1-1] | 1 [1-1] | 1 [1-2] |
| Pain | 1 [1-1] | 1 [1-1] | 1 [1-1] |
| Skin Irritation | 1 [1-1] | 1 [1-1] | 1 [1-2] |
| **Total number of complications reported** |  |  |  |
| Fever | 48 | 30 | 18 |
| Cough and Cold | 43 | 24 | 19 |
| Vomiting | 22 | 10 | 12 |
| Pain | 21 | 15 | 6 |
| Skin Irritation | 14 | 7 | 7 |
