# Supplementary material for: Hybrid Email and Outpatient Clinics to Optimize Maintenance Therapy in Acute Lymphoblastic Leukemia
Source: J Pediatr Hematol Oncol. 2023 Dec 12;46(1):39–45. doi: 10.1097/MPH.0000000000002796 (PMC10756697; doi:10.1097/MPH.0000000000002796)
Supplement: Supplementary file 9 [file mph-46-039-s009.docx]

| **SDC 9** |  |  |  |  |
| --- | --- | --- | --- | --- |
| **(a)** Distance matrices analysis for the cohort | |  |  |  |
|  | **Era 1** | **Era 2** | **Era 3** | **Era 4** |
|  |  |  |  | Completed MT |
| **N** | 135 | 90 | 48 | 59 |
| Direct | 111 | 54 | 30 | 32 |
| Virtual | 24* | 36 | 18 | 27 |
| **Minimum distance travelled for one visit one way (km)** |  |  |  |  |
| Overall | 66 [25-171] | 34 [18 - 181] | 125 [41 - 331] | 147 [29 - 296] |
| Direct | 52 [23-138] | 23 [16 - 44] | 99 [42 - 192] | 48 [20 - 250] |
| Virtual | 131 [53-332] | 181 [37 - 325] | 230 [41 - 361] | 186 [93 - 353] |
| Values represent Median [Interquartile range] unless stated otherwise | | | | |
| "Direct" patients are defined as patients who have fewer or an equal number of e-clinic consultations than the median e-clinic consultations/patient for the respective era | | | | |
| "Virtual" patients are defined as patients who have more e-clinic consultations than the median e-clinic consultations/patient for that era | | | | |
|  |  |  |  |  |
| **(b)** Distance matrices analysis for the cohort residing within and outside 100 km from TMC | | | | |
|  | **Era 1** | **Era 2** | **Era 3** | **Era 4** |
|  |  |  |  | Completed MT |
| **Patients residing within 100 km radius TMC (n, %)** | 84 (62) | 61 (68) | 22 (46) | 25 (42) |
| Direct | 73 (87) | 48 (79) | 15 (68) | 18 (72) |
| Virtual | 11 (13) | 13 (21) | 7 (32) | 7 (28) |
| **Patients residing outside 100 km radius TMC (n, %)** | 51 (38) | 29 (32) | 26 (54) | 34 (58) |
| Direct | 38 (75) | 6 (21) | 15 (58) | 14 (41) |
| Virtual | 13 (25) | 23 (79) | 11 (42) | 20 (59) |
| "Direct" patients are defined as patients who have fewer or an equal number of e-clinic consultations than the median e-clinic consultations/patient for the respective era | | | | |
| "Virtual" patients are defined as patients who have more e-clinic consultations than the median e-clinic consultations/patient for that era | | | | |
