# Supplementary material for: Hybrid Email and Outpatient Clinics to Optimize Maintenance Therapy in Acute Lymphoblastic Leukemia
Source: J Pediatr Hematol Oncol. 2023 Dec 12;46(1):39–45. doi: 10.1097/MPH.0000000000002796 (PMC10756697; doi:10.1097/MPH.0000000000002796)
Supplement: Supplementary file 10 [file mph-46-039-s010.docx]

| **SDC 10**. **(a)** Cost incurred during MT for patients who undertook survey | | |
| --- | --- | --- |
|  |  |  |
| **Survey results (Cost)** | **Reponses** |  |
| One-way OPD travel to TMC |  |  |
| 0-500 | 44 |  |
| 501-1000 | 38 |  |
| 1001-1500 | 41 |  |
| 1501-2000 | 9 |  |
| 2001 and above | 24 |  |
| **Email service** |  |  |
| Travel |  |  |
| 0 | 102 |  |
| 1-500 | 48 |  |
| 501-1000 | 3 |  |
| 1001-1500 | 2 |  |
| 1501 and above | 1 |  |
| Blood Test |  |  |
| 0-200 | 2 |  |
| 201-400 | 104 |  |
| 401-600 | 41 |  |
| 601-800 | 8 |  |
| 801 and above | 1 |  |
| Cost for e-mailing blood report |  |  |
| 0 | 135 |  |
| 1-50 | 18 |  |
| 51-100 | 3 |  |
|  |  |  |
| **(b)** Cost of one visit (OP vs E-mail) for patients who undertook survey | | |
|  | **OP Cost** | **E-mail** |
| Complete Blood Counts Report | 410* | 300 |
| Travel (to and fro) | 500 | 0 |
| Consultation | 150* | - |
| Sending E-mail | - | 0 |
| **Total** | 1060 | 300 |
| Note: Cost (in INR) refers to average cost (middle value) stated by majority of the families during survey; *Standard cost at Tata Medical Center Kolkata | | |
| **(c)** Total monetary saving with current MT mode of delivery as compared to only OP visits | | |
|  | **Cost** |  |
| Onsite (48 visits) | 50880 | (48*1060) |
| Current Practice (1/3 Onsite visit) + 32 e-mail | 26560 | ([50880/3] +32*300) |
| **Cost Saved** | 24320 |  |
| Note: Cost from Table SDC 10b is considered for calculations | | |
